# Molecular frequency of *Coxiella burnetii*, *Tropheryma whipplei*, *Brucella*, and *Bartonella* in heart valve tissues from patients with infective endocarditis: A cross-sectional study from Tehran, Iran

**DOI:** 10.1016/j.nmni.2026.101800

**Published:** 2026-06-19

**Authors:** Arezoo Dehghan, Mohammad Hossein Ahmadi, Saber Esmaeili, Mohammad Ali Boroumand, Mahshid Hesami

**Affiliations:** a Department of Microbiology, Faculty of Medicine, Shahed University, Tehran, Iran; b WHO Collaborating Centre for Vector-Borne Diseases, Department of Epidemiology and Biostatistics, Research Centre for Emerging and Reemerging Infectious Diseases, Pasteur Institute of Iran, Tehran, Iran; c National Reference Laboratory of Plague, Tularemia and Q Fever, Research Centre for Emerging and Reemerging Infectious Diseases, Pasteur Institute of Iran, Kabudar-Ahang, Akanlu, Hamadan, Iran; d Department of Pathology and Laboratory Medicine, Tehran Heart Center, Tehran University of Medical Sciences, Tehran, Iran; e Cardiogenetic Research Center, Rajaie Cardiovascular Institute, Tehran, Iran

**Keywords:** Infective endocarditis, Blood culture-negative endocarditis, Molecular diagnosis, *Brucella*, *Bartonella*, *Coxiella burnetii*, *Tropheryma whipplei*

## Abstract

**Background:**

Infective endocarditis (IE) is a life-threatening condition caused by diverse microbial agents and remains a major diagnostic and therapeutic challenge, particularly in cases of blood culture–negative IE (BCN-IE).

**Methods:**

In this cross-sectional study, 405 formalin-fixed paraffin-embedded heart valve tissue samples from 232 patients with pathologically and clinically confirmed IE who underwent valve replacement were collected between March 2021 and March 2025 from two tertiary referral hospitals in Tehran, Iran. Following DNA extraction, *Coxiella burnetii*, *Brucella* spp., and *Bartonella* spp. were detected using TaqMan real-time PCR, while *Tropheryma whipplei* was identified by SYBR Green real-time PCR. Species-level identification of *Brucella* and *Bartonella* in positive samples was performed using conventional and multiplex real-time PCR.

**Results:**

Overall, *C. burnetii*, *Brucella* spp., *Bartonella* spp., and *T. whipplei* were detected in 6.9%, 8.6%, 5.6%, and 3.0% of patients, respectively. The study population comprised 156 males (67.2%) and 76 females (32.8%), with a mean age of 47 years. The aortic (40.3%) and mitral (39.1%) valves were the most frequently involved sites. *Bartonella quintana*, *Brucella melitensis*, and *Brucella abortus* were identified among positive samples. Dyspnea was significantly associated with *C. burnetii* (P = 0.02), *Bartonella* (P = 0.04), and *Brucella* infections (P = 0.009), while fever (P = 0.03) and sweating (P = 0.001) were significantly associated with *Brucella* infection.

**Conclusions:**

Fastidious and intracellular pathogens are important but overlooked causes of BCN-IE in Iran. Molecular analysis of heart valve tissues represents a valuable diagnostic strategy and supports the need for improved surveillance, prevention, and targeted management of zoonotic and fastidious agents in IE.

## Highlights

• Molecular analysis revealed fastidious pathogens in heart valve tissues of infective endocarditis patients.
• *Coxiella burnetii*, *Brucella* spp., *Bartonella* spp., and *Tropheryma whipplei* were detected in culture-negative cases.
• Aortic and mitral valves were the most frequently involved cardiac sites.
• Dyspnea, fever, and sweating showed significant associations with selected zoonotic infections.
• Molecular testing of excised valves enabled more accurate etiological diagnosis of blood culture–negative endocarditis.

## 1 Introduction

Infective endocarditis (IE) is a serious and potentially fatal condition characterized by infection and inflammation of the endocardium and heart valves [1,2]. Endocarditis as a rare but high mortality disease is generally classified as infectious and non-infectious (NIE) [3].

In approximately 80% of IE cases, the causative pathogens are conventional bacteria, including *Streptococcus*, *Staphylococcus*, and *Enterococcus* species. However, nearly 20% of cases are attributed to fastidious or atypical microorganisms such as members of the HACEK group, *Coxiella burnetii*, *Bartonella* spp., *Brucella* spp., *Tropheryma whipplei*, and *Legionella* spp., which frequently result in blood culture–negative infective endocarditis (BCN-IE) [1,4].

The pathogenesis of IE begins with microbial adhesion to damaged endothelial surfaces, followed by bacterial colonization, activation of inflammatory and cytokine pathways, increased fibronectin deposition, and subsequent formation of vegetative thrombotic lesions. Risk factors such as prior cardiac surgery, prosthetic valves, hemodialysis, injection drug use, and intravascular catheterization significantly increase susceptibility to the infection [4,5].

Despite advances in antimicrobial therapy and diagnostic modalities, the global burden of IE has risen over recent decades, with increasing incidence and mortality rates, particularly among older male populations [4,6].

According to the modified Duke criteria and European Society of Cardiology (ESC) guidelines, microbiological evidence and cardiac imaging remain central to the diagnosis of IE. However, conventional blood cultures fail to identify the causative agent in up to 20% of cases, underscoring the importance of serological and nucleic acid–based diagnostic approaches as rapid and accurate methods, particularly for fastidious intracellular pathogens [[6], [7], [8]].

*Coxiella burnetii*, an obligate intracellular Gram-negative bacterium and the causative agent of Q fever, represents the most frequent etiological agent of BCN-IE worldwide [9,10]. *Bartonella* spp., particularly *B. quintana* and *B. henselae*, account for a substantial proportion of BCN-IE through biofilm-associated valvular infection [11]. *Brucella* spp., zoonotic facultative intracellular coccobacilli, represent the leading cause of mortality in brucellosis and are responsible for a small but clinically significant fraction of BCN-IE cases [12]. *Tropheryma whipplei*, the causative agent of Whipple's disease, is an emerging pathogen in culture-negative endocarditis, often diagnosed only through molecular analysis of excised heart valve tissue [13].

Despite the recognized role of fastidious pathogens in blood culture–negative infective endocarditis, molecular data from Iran remain scarce and largely limited to single-agent studies. This study addresses this gap by providing a comprehensive molecular assessment of *C*. *burnetii*, *T*. *whipplei*, *Brucella* spp., and *Bartonella* spp. in heart valve tissues from patients with confirmed infective endocarditis in major referral centers in Iran. By simultaneously targeting multiple key etiological agents, this work offers novel epidemiological insights and underscores the clinical value of molecular diagnostics for improving the etiological identification and management of infective endocarditis in endemic settings.

## 2 Material and methods

### 2.1 Study design and sample collection

This cross-sectional study included 405 formalin-fixed paraffin-embedded (FFPE) heart valve tissue blocks obtained from 232 patients with clinically and pathologically confirmed infective endocarditis who underwent valve replacement surgery. Samples were collected between March 2021 and March 2025 from two tertiary cardiac referral centers in Tehran, Iran: Shahid Rajaee Heart Center and Tehran Heart Center. Clinical and pathological records were reviewed to confirm the diagnosis and eligibility criteria. Selected samples were transferred to the Pasteur Institute of Iran (National Reference Laboratory) for molecular analysis.

### 2.2 Sectioning and deparaffinization of FFPE tissues

Paraffin blocks were sectioned into 5-μm-thick slices using a sterile scalpel blade and transferred into labeled sterile microtubes. Deparaffinization was performed by adding 1200 μL of xylene to each tube, followed by vortexing for 5 min and incubation in a thermomixer at 70 °C for 10 min. Samples were then centrifuged at 13000 rpm for 5 min, and the supernatant was carefully removed. Subsequently, 1200 μL of absolute ethanol was added, vortexed for 1–2 min, and centrifuged at 13000 rpm for 5 min. The ethanol washing step was repeated twice, followed by air-drying of the tissue pellet [[14], [15], [16]].

### 2.3 DNA extraction and quality assessment

All tissue samples were homogenized using a TissueLyser (Qiagen, Germany) for 6 min at 30 Hz, before starting the DNA extraction process. Genomic DNA was extracted from deparaffined tissues samples using a commercial DNA Extraction Kit (Viragene Co, Iran), according to the manufacturer's instructionsDNA concentration and purity were assessed using a NanoDrop spectrophotometer (Epoch, BioTek, USA). Samples with A260/A280 ratios between 1.8 and 2.0 and A260/A230 ratios between 2.0 and 2.5 were considered suitable for downstream molecular analyses.

### 2.4 Molecular detection of *Coxiella burnetii*

All extracted DNA samples were screened for *C. burnetii* by TaqMan real-time PCR targeting the IS1111 gene (Table 1). Amplification was performed using the Rotor-Gene 6000 real-time PCR system (Corbett, Victoria, Australia) with the following cycling conditions: initial activation at 95 °C for 3 min, followed by 45 cycles of denaturation at 95 °C for 10 s and annealing/extension at 60 °C for 30 s. Data were analyzed using Rotor-Gene® Q software version 2.3.5 (QIAGEN).

**Table 1:** Primer and probe sequences used in the molecular methods.

Pathogen	Gene	F-primer (5′-3′)	R-primer (5′-3′)	Product Size (bp)	Reference
*Coxiella burnetii*	IS1111	AAAACGGATAAAAAGAGTCTGTGGTT	CCACACAAGCGCGATTCAT	70	[17,18]
	Probe	6-FAM-AAAGCACTCATTGAGCGCCGCG-TAMRA			
Brucella	IS711	GCTTGAAGCTTGCGGACAGT	GGCCTACCGCTGCGAAT	63	[18,19]
	Probe	FAM-AAGCCAACACCCGGCCATTATGGT-TAMRA			
Bartonella	16s-23srRNA	GGGGAAGGTTTTCCGGTTTATC	GAGGACTTGAACCTCCGACC	92	[20]
	Probe	6_FAM_GGAGGGCTTGTAGCTCAGYTGGTTAGAGCG-TAMRA			
Bartonella	16srRNA	TTAGAGTGAGCGGCAAAC	TACCGTCATTATCTTCACCG	356	[21]
*Tropheryma whipplei*	TWSP	AGAGAGATGGGGTGCAGGAC	AGCCTTTGCCAGACAGACAC	210	[22]
*Brucella melitensis*	BMEII0466	TCGCATCGGCAGTTTCAA	CCAGCTTTTGGCCTTTTCC	67	[19,23]
	Probe	TEX-CCTCGGCATGGCCCGCAA-BHQ			
*Brucella abortus*	BruAb2_0168	GCACACTCACCTTCCACAACAA	CCCCGTTCTGCACCAGACT	81	[19,23]
	Probe	HEX-TGGAACGACCTTTGCAGGCGAGATC-BHQ−1			
*Brucella suis*	BR0952	CCTGCAAAAAGCAGGAACCA	CCTCCGCCAGTCGTGAAA	965	[19]
*Brucella canis*	BMEII0635-0636	AAAATGCGGATCGGCCTT	TCCCGGCGCATTGCT	749	[19]

### 2.5 Molecular identification of *Bartonella* spp.

Detection of *Bartonella* spp. was carried out using real-time PCR targeting the 16S–23S rRNA intergenic spacer region (Table 1). The amplification protocol consisted of an initial activation at 95 °C for 3 min, followed by 45 cycles of 95 °C for 10 s and 60 °C for 30 s, using the Rotor-Gene 6000 system.

All *Bartonella*-positive samples were further analyzed by conventional PCR targeting the 16S rRNA gene for identification of *Bartonella* species (Table 1). PCR products were visualized on a 1.5% agarose gel, and samples yielding specific bands were subjected to Sanger sequencing. Obtained sequences were analyzed using BLAST against the NCBI GenBank database (https://blast.ncbi.nlm.nih.gov/Blast.cgi) and MEGA11 software, and phylogenetic analysis was performed.

### 2.6 Molecular identification of *Brucella* spp.

TaqMan real-time PCR targeting the IS711 gene was used for the detection of *Brucella* spp. in all DNA samples (Table 1). The amplification program consisted of an initial activation at 95 °C for 3 min, followed by 45 cycles of 95 °C for 10 s and 60 °C for 30 s, using the Rotor-Gene 6000 system.

Species-level identification of *Brucella*-positive samples was performed using multiplex real-time PCR targeting specific genes of *Brucella melitensis* and *Brucella abortus*. Additionally, conventional PCR assays were conducted to detect *Brucella suis* and *Brucella canis* (Table 1), with amplification products analyzed by electrophoresis on 2% agarose gels [19].

### 2.7 Molecular detection of *Tropheryma whipplei*

Detection of *T. whipplei* was performed using SYBR Green real-time PCR targeting the *wisp* gene (Table 1). Amplification conditions included an initial activation at 95 °C for 15 min, followed by 45 cycles of denaturation at 95 °C for 15 s, annealing at 59 °C for 30 s, and extension at 72 °C for 30 s. After amplification, the melting protocol was followed by raising the temperature slowly from 65 to 97 °C to differentiate the specific and non-specific amplified amplicons. The samples with suitable amplification and melting (80 ± 0.5 °C) curves were considered positive for *T. whipplei*.

### 2.8 Statistical analysis

Statistical analyses were performed using SPSS software version 27.0 (IBM Corp., Armonk, NY, USA). Descriptive statistics were used to summarize demographic, clinical, and laboratory data. Categorical variables were expressed as frequencies and percentages, while continuous variables were reported as mean ± standard deviation or median with interquartile range, as appropriate. The frequency of each detected pathogen was calculated with corresponding proportions. Comparisons between categorical variables were conducted using the chi-square test or Fisher's exact test, when applicable. A two-tailed *P* value of <0.05 was considered statistically significant.

## 3 Results

### 3.1 Demographic, clinical, and laboratory characteristics

A total of 405 formalin-fixed paraffin-embedded heart valve tissue samples (131 patients with 1 block, 64 with 2 blocks, 23 with 3 blocks, 18 with 4 blocks and 1 patient with 5 blocks) obtained from 232 patients with infective endocarditis were included in this study. The study population comprised 156 males (67.2%) and 76 females (32.8%), with a mean age of 47.3 ± 20 years. Patients originated from 25 provinces across Iran, with the highest number of cases reported from Tehran (48.0%, n = 110), followed by Gilan (8.3%, n = 19) and Alborz (7.8%, n = 18) (Table 2, Table 3).

**Table 2:** Summary results of frequency analyses.

Characteristics	All Patient of IE (%)	*C. burnetii* positive (%)	*Bartonella* positive (%)	*Brucella* positive (%)	*T*. *whipplei* positive (%)
Female	76 (33)	6 (37.5)	2 (15.4)	9 (45)	3 (43)
Male	156 (67)	10 (62.5)	11 (84.6)	11 (55)	4 (57)
Age (mean)	47.3	44	49	45	51
Village	12 (5)	3 (18.8)	0	1 (5)	0
Urban	220 (95)	13 (81.2)	13 (100)	19 (95)	7 (100)
BCN	159 (68.5)	13 (81.2)	9 (69)	19 (95)	7 (100)
TCN	124 (53)	7 (43.8)	7 (53.8)	17 (85)	6 (85.7)
Death	43 (18.5)	5 (31.2)	3 (23)	4 (20)	0
Mitral valve	99 (38.8)	6 (33.3)	8 (53.34)	5 (25)	1 (12.5)
Aortic valve	102 (40)	11 (61.2)	5 (33.34)	11 (55)	6 (75)
Tricuspid	23 (9)	1 (5.55)	1 (6.66)	0	0
Pulmonary	8 (3.2)	0	1 (6.66)	1 (5)	1 (12.5)
Pericardium wall	23 (9)	0	0	3 (15)	0
Total Number of patients	232	16	13	20	7
Total Number of tissues	255	18	15	20	8

IE: infective endocarditis; BCN: Blood culture negative; TCN: tissue culture negative.

**Table 3:** Province distribution of patients.

Province	Total (%)	*C*. *burnetii* Positive (%)	*Bartonella* Positive (%)	*Brucella* Positive (%)	*T. whipplei* Positive (%)
Ardabil	5 (2.1)	0	0	0	0
East Azarbaijan	1 (0.43)	0	0	0	0
West Azarbaijan	2 (0.86)	0	0	1 (5)	0
Tehran	110 (47.4)	7 (43.8)	8 (61.5)	10 (50)	4 (57)
Khorasan Razavi	1 (0.43)	0	0	0	0
Khozestan	9 (3.87)	0	0	0	0
Zanjan	2 (0.86)	0	0	0	1 (14)
Semnan	2 (0.86)	0	0	0	0
Sistan and Baluchestan	4 (1.72)	1 (6.3)	0	0	0
Chaharmahal and Bakhtiari	1 (0.43)	0	0	0	0
Qazvin	3 (1.3)	1 (6.3)	0	2 (10)	0
Qom	7 (3)	0	1 (7.7)	0	0
Isfahan	4 (1.72)	0	0	1 (5)	0
Alborz	18 (7.75)	1 (6.3)	1 (7.7)	1 (5)	0
Kurdistan	3 (1.3)	0	0	0	0
Kerman	3 (1.3)	1 (6.3)	0	1 (5)	0
Kermanshah	4 (1.72)	1 (6.3)	0	1 (5)	0
Golestan	5 (2.1)	0	1 (7.7)	0	0
Gilan	19 (8.2)	2 (12.5)	1 (7.7)	1 (5)	1 (14)
Lorestan	6 (2.6)	1 (6.3)	1 (7.7)	0	0
Mazandaran	9 (3.87)	0	0	1 (5)	1 (14)
Markazi	3 (1.3)	0	0	1 (5)	0
Hormozgan	3 (1.3)	1 (6.3)	0	0	0
Hamedan	4 (1.72)	0	0	0	0
unknown	3 (1.3)	0	0	0	0
Other nationalities (Afghanistan)	1 (0.43)	0	0	0	0
Total	232	16	13	20	7

Blood and tissue cultures were performed for 209 and 162 samples, respectively. Positive results were obtained in 50 blood cultures and 38 tissue cultures, while the remaining cultures were negative. The most frequently isolated organisms from culture-positive samples were *Streptococcus* spp., *Staphylococcus* spp., *Enterococcus* spp., and *Pseudomonas aeruginosa*. Among the 232 patients, the number of tissue samples obtained from the aortic, mitral, tricuspid, and pulmonary valves, as well as from pericardial tissue, was 102, 99, 23, 8, and 23, respectively. Regarding the anatomical distribution of samples, 40.0% originated from the aortic valve, 38.8% from the mitral valve, 9.0% from the tricuspid valve, 3.2% from the pulmonary valve, and 9.0% from pericardium tissue, among a total of 255 tissue samples obtained from 232 patients.

Fever (≥38 °C) was documented in 16.0% of patients. Diabetes mellitus, hypertension, and substance use were present in 8.0%, 15.0%, and 9.0% of cases, respectively. The most common presenting symptoms included dyspnea (42.2%), chest pain (16.5%), fever (16%), and unintentional weight loss. Overall, 43 patients (18.5%) died due to cardiac complications (Table 4).

**Table 4:** Analysis of factors related to *C*. *burnetii*, *T*. *whipplei*, *Brucella* and *Bartonella* in patients with infective endocarditis from 2021 to 2025.

Characteristics		*C. burnetii* Positive			*Bartonella* spp. Positive			*Brucella* spp. Positive			*T. whipplei* Positive		
		Count (%)	P value	OR (CI)	Count (%)	P value	OR (CI)	Count (%)	P value	OR (CI)	Count	P value	OR (CI)
Gender	Female	6 (37.5)	0.6	0.8 (0.28-2.3)	2 (15.4)	0.2	2.8 (0.6-13)	9 (45)	0.2	0.56 (0.2-1.4)	3 (42.9)	0.68	0.64 (0.14-2.9)
	Male	10 (62.5)			11 (84.6)			11 (55)			4 (57.1)		
Death	No	11 (68.8)	0.18	2 (0.7-6.5	10 (76.9)	0.7	1.3 (0.35-5)	16 (80)	0.7	1.1 (0.34-3.4)	6 (85.7)	0.35	0.96 (0.93-0.99)
	Yes	5 (31.3)			3 (23.1)			4 (20)			1 (14.3)		
Smoking	Negative	15 (93.8)	0.99	0.65 (0.08-5)	10 (76.9)	0.1	3.3 (0.8-13)	20 (100)	0.22	0.9 (0.86-0.94)	6 (85.7)	0.49	1.7 (0.19-14.9)
	Positive	1 (6.3)			3 (23.1)			0			1 (14.3)		
Diabetic	Negative	15 (93.8)	0.99	0.7 (0.09-5.8)	10 (76.9)	0.08	3.8 (0.95-15)	20 (100)	0.38	0.9 (0.86-0.4	6 (85.7)	0.45	1.9 (0.2-16)
	Positive	1 (6.3)			3 (23.1)			0			1 (14.3)		
High blood pressure	Negative	14 (87.5)	0.99	0.8 (0.18-3.8)	10 (76.9)	0.4	1.8 (0.47-7)	19 (95)	0.3	0.28 (0.037-2.2)	5 (71.4)	0.99	0.97 (0.11-8.3)
	Positive	2 (12.5)			3 (23.1)			1 (5)			2 (28.6)		
Dyspnea	Negative	5 (31.3)	0.02	3.2 (1-9.7)	4 (30.8)	0.04	3 (0.98-11)	6 (30)	0.009	3.5 (1.3-9.6)	3 (42.9)	0.99	1 (0.22-4.6)
	Positive	11 (68.8)			9 (69.2)			14 (70)			4 (57.1)		
Chest pain	Negative	15 (93.8)	0.48	0.3 (0.04-2.5)	12 (92.3)	0.7	0.4 (0.05-3.2)	18 (90)	0.54	0.54 (0.12-2.44)	3 (42.9)	0.1	7.4 (1.6-34.9)
	Positive	1 (6.3)			1 (7.7)			2 (10)			4 (57.1)		
Fever	Negative	12 (75)	0.29	1.8 (0.56-6)	9 (69.2)	0.13	2.5 (0.7-8.6)	11 (55)	0.001	5.3 (2-14)	7 (100)	0.6	0.96 (0.94-0.99)
	Positive	4 (25)			4 (30.8)			9 (45)			0		
Chills	Negative	13 (81.3)	0.47	1.4 (0.4-5.5)	9 (69.2)	0.08	3 (0.87-10.5)	13 (65)	0.1	4 (1.46-11)	7 (100)	0.59	0.96 (0.94-0.99)
	Positive	3 (18.8)			4 (30.8)			7 (35)			0		
Fatigue	Negative	15 (93.8)	0.99	0.8 (0.097-6)	10 (76.9)	0.07	4 (1-16.4)	18 (90)	0.65	1.36 (0.3-6.3)	5 (71.4)	0.09	5 (0.93-29)
	Positive	1 (6.3)			3 (23.1)			2 (10)			2 (28.6)		
Weight loss	Negative	16 (100)	0.99	0.9 (0.8-0.96)	13 (100)	0.99	0.94 (0.9-0.97)	18 (90)	0.27	2.2 (0.45-11)	6 (85.7)	0.3	3.2 (0.35-29)
	Positive	0			0			2 (10)			1 (14.3)		
Palpitation	Negative	15 (93.8)	0.58	1.2 (0.15-10)	12 (92.3)	0.5	1.5 (0.18-13)	19 (95)	0.99	0.96 (0.11-7.8)	7 (100)	0.99	0.96 (0.94-0.99)
	Positive	1 (6.3)			1 (7.7)			1 (5)			0		
Sweating	Negative	16 (100)	0.99	0.9 (0.89-0.96)	13 (100)	0.99	0.94 (0.91-0.97)	17 (85)	0.03	6 (1.4-26)	7 (100)	0.99	0.96 (0.94-0.99)
	Positive	0			0			3 (15)			0		
Cough	Negative	15 (93.8)	0.5	1.3 (0.16-11.4)	11 (84.6)	0.12	4 (0.8-22)	17 (85)	0.058	4.5 (1-18)	7 (100)	0.99	0.96 (0.94-0.99)
	Positive	1 (6.3)			2 (15.4)			3 (15)			0		
Edema	Negative	16 (100)	0.99	0.9 (0.9-0.96)	12 (92.3)	0.3	2.9 (0.3-26.5)	20 (100)	0.99	0.9 (0.87-0.94)	7 (100)	0.99	0.96 (0.94-0.99)
	Positive	0			1 (7.7)			0			0		
Location	Urban	13 (81.3)	0.04	5 (1.2-22)	13 (100)	0.99	0.94 (0.9-0.97	19 (95)	0.99	0.96 (0.11-7.8)	7 (100)	0.99	0.96 (0.94-0.99)
	Village	3 (18.8)			0			1 (5)			0		
Valve type	Aortic	11 (61.2)	0.05	2.5 (1-6.7)	5 (33.34)	0.58	0.0.73 (0.24-2.2)	11 (55)	0.15	1.9 (0.77-4.8)	6 (75)	0.05	4.7 (1-24)
	Mitral	6 (33.3)	0.62	0.77 (0.3-2)	8 (53.34)	0.2	1.88 (0.65-5.3)	5 (25)	0.18	0.5 (0.17-1.4)	1 (12.5)	0.15	0.2 (0.026-1.8)
	Pulmonary	0	0	0	1 (6.66)	0.99	2.4 (0.27-20.6)	1 (5)	0.48	1.7 (0.2-14.6)	1 (12.5)	0.22	4.9 (0.5-45.4)
	Tricuspid	1 (5.55)	0.99	0.57(0.07-4.5)	1 (6.66)	0.99	0.7 (0.09-5.6)	0	0	0	0	0	0
	Pericardium tissue	0	0	0	0	0	0	3 (15)	0.4	1.9 (0.5-7)	0	0	0

OR: odds ratio; CI: confidence interval.

### 3.2 Detection of *Coxiella burnetii*

*Coxiella burnetii* DNA was detected in 16 of 232 patients (6.9%). Of the positive cases, 11 samples were obtained from Shahid Rajaee Heart Center and five from Tehran Heart Center. Most affected patients were male (n = 10), with a mean age of 44 years. Aortic valve involvement was the most frequent finding (61.2%) and was significantly associated with *C. burnetii* positivity (odds ratio [OR]: 2.5; 95% confidence interval [CI]: 1–6.7; P = 0.05). The majority of positive cases were residents of Tehran Province, and five patients (31.3%) died during hospitalization (Table 5).

**Table 5:** Clinical characteristics of patients infected by *Coxiella burnetii* and *Bartonella* spp.

***Coxiella burnetii***	**Patients ID**	**Valve**	**Province**	**Gender**	**Blood Group**	**Age**	**Death**	**Smoking**	**Diabetic**	**High Blood Pressure**	**Dyspnea**	**Chest pain**	**Edema**	**Fever**	**Chills**	**Fatigue**	**Weight loss**	**Palpitation**	**Sweating**	**Cough**	**Location**
	E251	Aortic	Karaj	Female	O+	31	Yes	No	No	No	Yes	No	No	No	No	Yes	No	No	No	No	Urban
	E323	Mitral	Tehran	Male	O+	12		No	No	No	No	No	No	Yes	Yes	No	No	No	No	No	Urban
	E274	Tricuspid	Gilan	Female	AB+	57		No	No	No	Yes	No	No	No	No	No	No	No	No	No	Village
	E255	Tricuspid	Tehran	Female	A+	64	Yes	No	No	Yes	Yes	No	No	No	No	No	No	No	No	No	Urban
	E374	Tricuspid	Sistan and Baluchestan	Female	A+	19	Yes	No	No	No	Yes	No	No	Yes	Yes	No	No	No	No	No	Village
	E325	Tricuspid & Aortic	Kermanshah	Male	B+	36		No	No	No	Yes	No	No	No	No	No	No	No	No	No	Urban
	E445	Tricuspid	Tehran	Male	B+	42		No	No	No	No	Yes	No	No	No	No	No	No	No	No	Urban
	E287	Mitral	Hormozgan	Female	A+	53		No	No	No	No	No	No	No	No	No	No	No	No	No	Village
	E262	Mitral	Qazvin	Female	A+	37		No	No	No	Yes	No	No	Yes	Yes	No	No	No	No	No	Urban
	E278	Mitral & Aortic	Tehran	Male	O+	56		No	No	No	Yes	No	No	No	No	No	No	No	No	No	Urban
	E464	Aortic	Tehran	Male	A+	25		Yes	No	No	Yes	No	No	No	No	No	No	No	No	No	Urban
	E394	Mitral	Lorestan	Male	A+	66	Yes	No	Yes	Yes	Yes	No	No	No	No	No	No	No	No	Yes	Urban
	E451	Aortic	Tehran	Male	A+	71	Yes	No	No	No	Yes	No	No	No	No	No	No	No	No	No	Urban
	E288	Aortic	Kerman	Male	B+	31		No	No	No	No	No	No	Yes	No	No	No	No	No	No	Urban
	E254	Mitral	Tehran	Male	A+	62		No	No	No	Yes	No	No	No	No	No	No	Yes	No	No	Urban
	E453	Aortic	Gilan	Male	B+	42		No	No	No	No	No	No	No	No	No	No	No	No	No	Urban
***Bartonella* spp.**	E251	Aortic	Karaj	Female	O+	31	Yes	No	No	No	Yes	No	No	No	No	Yes	No	No	No	No	Urban
	E469	Aortic & Tricuspid	Tehran	Male	A+	64		No	Yes	No	Yes	No	No	No	No	No	No	No	No	No	Urban
	E419	Mitral	Tehran	Male	O+	43		Yes	No	No	Yes	No	No	No	No	No	No	No	No	No	Urban
	E313	Aortic	Tehran	Male	B+	63		No	Yes	Yes	Yes	No	Yes	No	No	No	No	No	No	No	Urban
	E465	Mitral	Tehran	Male	O+	31		Yes	No	No	Yes	No	No	Yes	Yes	No	No	No	No	Yes	Urban
	E270	Mitral & Aortic	Tehran	Male	A+	56		No	No	No	Yes	No	No	No	No	No	No	No	No	No	Urban
	E268	Mitral	Tehran	Male	O-	78	Yes	No	No	No	No	No	No	No	No	No	No	No	No	No	Urban
	E395	Aortic	Golestan	Male	A+	34		Yes	No	No	Yes	No	No	Yes	Yes	No	No	No	No	No	Urban
	E394	Mitral	Lorestan	Male	A+	66	Yes	No	Yes	Yes	Yes	No	No	No	No	No	No	No	No	Yes	Urban
	E458	Mitral	Gilan	Male	B+	67		No	No	Yes	No	Yes	No	No	No	No	No	No	No	No	Urban
	E309	Pulmonary	Qom	Female	O+	22		No	No	No	No	No	No	Yes	Yes	Yes	No	No	No	No	Urban
	E254	Mitral	Tehran	Male	A+	62		No	No	No	Yes	No	No	No	No	No	No	Yes	No	No	Urban
	E240	Mitral	Tehran	Male	B-	31		No	No	No	No	No	No	Yes	Yes	Yes	No	No	No	No	Urban

### 3.3 Detection and species identification of *Bartonella spp*.

*Bartonella* spp. were detected in 13 patients (5.6%). Six positive samples were obtained from Shahid Rajaee Heart Center and seven from Tehran Heart Center. The majority of affected patients were male (n = 10), with a mean age of 49 years. Mitral valve involvement was more frequent (53.34%); however, this association did not reach statistical significance (OR: 1.88; 95% CI: 0.65–5.3; P = 0.2). Most cases were from Tehran province, and three patients (23.1%) died (Table 5).

**Fig. 1:**
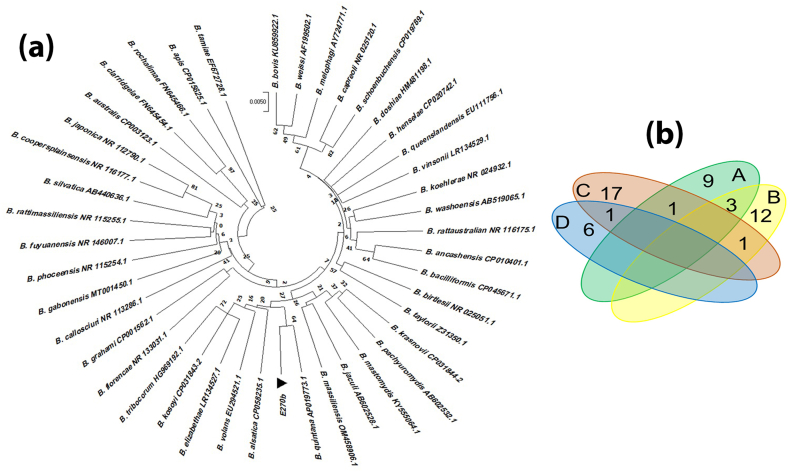
(a): Phylogenetic tree based on *16S rRNA* gene sequences of *Bartonella* spp., showing the relationship between reference strains and the study isolate E270b, which clusters within the *Bartonella quintana* lineage. (b): Venn diagram illustrating single and co-infections with *Coxiella burnetii, Brucella spp., Bartonella spp.,* and *Tropheryma whipplei* detected by molecular analysis of heart valve tissues from patients with infective endocarditis. A: *Bartonella; B: Coxiella burnetii;* C: *Brucella;* D: *Tropheryma whipplei*.

Sequencing analysis confirmed *Bartonella quintana* in one case (the isolate E270b). The corresponding *16S rRNA* gene sequence was deposited in the NCBI GenBank database under accession number PX675817 (https://www.ncbi.nlm.nih.gov/nuccore/PX675817). Fig. 1a presents the phylogenetic analysis of *Bartonella* spp., demonstrating that isolate E270b clusters within the *B*. *quintana* clade.

### 3.4 Detection and species identification of *Brucella spp.*

*Brucella* spp. DNA was detected in 20 patients (8.6%). Of these, 11 samples were from Shahid Rajaee Heart Center and five from Tehran Heart Center. Positive cases included 11 males and nine females, with a mean age of 45 years. The aortic valve (55%) was the most frequently involved one; however, this association did not reach statistical significance (OR: 1.9; 95% CI: 0.77–4.8; P = 0.15). Most patients originated from Tehran and Qazvin provinces, and four patients (20.0%) died (Table 6).

**Table 6:** Clinical characteristics of patients infected with *Brucella* spp. and *Trophryma whipplei*.

***Brucella* spp**	**Patients ID**	**Valve**	**Province**	**Gender**	**Blood Group**	**Age**	**Death**	**Smoking**	**Diabetic**	**High Blood Pressure**	**Dyspnea**	**Chest pain**	**Edema**	**Fever**	**Chills**	**Fatigue**	**Weight loss**	**Palpitation**	**Sweating**	**Cough**	**Location**
	E331	Aortic	Tehran	Male	O+	31		No	No	No	Yes	No	No	No	No	Yes	No	No	No	No	Urban
	E411	Aortic	Karaj	Female	AB+	52		No	No	Yes	Yes	No	No	Yes	No	No	No	Yes	No	No	Urban
	E324	Mitral	Tehran	Female	B+	71		No	No	No	No	No	No	Yes	Yes	No	No	No	No	No	Urban
	E415	Pericardium	Tehran	Female	AB+	50		No	No	No	Yes	No	No	No	Yes	No	No	No	No	Yes	Urban
	E266	Pulmonary	West Azarbaijan	Male	O+	31		No	No	No	Yes	No	No	Yes	No	No	No	No	No	No	Village
	E259	Mitral	Tehran	Male	AB+	52		No	No	No	Yes	No	No	Yes	Yes	No	No	No	Yes	No	Urban
	E258	Aortic	Tehran	Female	O+	4		No	No	No	No	No	No	No	No	No	No	No	No	No	Urban
	E261	Aortic	Kerman	Male	O+	46	Yes	No	No	No	Yes	No	No	Yes	Yes	No	No	No	No	No	Urban
	E256	Mitral	Kashan	Male	O-	22		No	No	No	No	No	No	No	No	No	No	No	No	No	Urban
	E414	Pericardium	Tehran	Female	AB+	69		No	No	No	Yes	No	No	Yes	No	No	No	No	No	No	Urban
	E410	Aortic	Qazvin	Female	B+	63		No	No	No	Yes	No	No	No	No	No	No	No	No	Yes	Urban
	E326	Aortic	Markazi	Male	B+	51		No	No	No	Yes	No	No	No	No	No	No	No	No	No	Urban
	E284	Aortic	Gilan	Female	AB+	29	Yes	No	No	No	Yes	No	No	No	No	No	No	No	No	No	Urban
	E262	Mitral	Qazvin	Female	A+	37		No	No	No	Yes	No	No	Yes	Yes	No	No	No	No	No	Urban
	E252	Aortic	Tehran	Female	O+	50	Yes	No	No	No	Yes	No	No	No	No	No	Yes	No	No	No	Urban
	E413	Pericardium	Kermanshah	Male	O+	88		No	No	No	Yes	No	No	No	No	No	No	No	No	Yes	Urban
	E408	Aortic	Tehran	Male	O+	44		No	No	No	No	Yes	No	No	No	No	No	No	Yes	No	Urban
	E257	Aortic	Tehran	Male	A+	27		No	No	No	No	Yes	No	No	No	No	Yes	No	No	No	Urban
	E240	Mitral	Tehran	Male	B-	31		No	No	No	No	No	No	Yes	Yes	Yes	No	No	No	No	Urban
	E253	Aortic	Mazandaran	Male	B+	67	Yes	No	No	No	Yes	No	No	Yes	Yes	No	No	No	Yes	No	Urban
***Trophryma whipplei***	E331	Aortic	Tehran	Male	O+	31		No	No	No	Yes	No	No	No	No	Yes	No	No	No	No	Urban
	E460	Mitral & Aortic	Tehran	Female	A+	41		No	No	No	No	Yes	No	No	No	Yes	Yes	No	No	No	Urban
	E355	Aortic	Mazandaran	Female	A+	64		No	No	Yes	Yes	No	No	No	No	No	No	No	No	No	Urban
	E407	Aortic	Tehran	Male	A-	63		No	Yes	Yes	Yes	Yes	No	No	No	No	No	No	No	No	Urban
	E431	Aortic	Tehran	Male	A+	53		Yes	No	No	No	Yes	No	No	No	No	No	No	No	No	Urban
	E418	Pulmonary	Gilan	Female	O+	40		No	No	No	Yes	No	No	No	No	No	No	No	No	No	Urban
	E289	Aortic	Zanjan	Male	O+	70		No	No	No	No	Yes	No	No	No	No	No	No	No	No	Urban

Species-level identification revealed *B*. *melitensis* in eight samples and *B*. *abortus* in one sample. No samples tested positive for *B*. *canis* or *B*. *suis*.

### 3.5 Detection of *Tropheryma whipplei*

*Tropheryma whipplei* was detected in seven patients (3.0%), including four males and three females, with a mean age of 51 years. The aortic valve (75%) was the predominant site of infection (OR:4.7; 95% CI: 1–24; P = 0.05). Most positive cases were from Tehran province, and no deaths were reported among patients with *T. whipplei*–associated infective endocarditis (Table 6).

### 3.6 Comparative analysis of detected pathogens

Among the investigated pathogens, *Brucella* spp. showed the highest molecular frequency (8.6%), followed by *C*. *burnetii* (6.9%), *Bartonella* spp. (5.6%), and *T*. *whipplei* (3.0%). Male predominance was observed in all groups. Aortic valve involvement was most frequent in *C. burnetii*, *Brucella* spp., and *T. whipplei* infections, whereas mitral valve involvement predominated in *Bartonella* spp. cases. Mortality was observed in patients with *C. burnetii*, *Brucella* spp., and *Bartonella* spp., while no deaths occurred among *T. whipplei*–positive patients. Most positive cases across all pathogen groups originated from Tehran province.

Dyspnea was identified as a significant risk factor across the analyzed pathogens, showing statistically significant associations with *C*. *burnetii* (OR: 3.2; 95% CI: 1–9.7; P = 0.02), *Bartonella* (OR: 3; 95% CI: 0.98–11; P = 0.04) and, *Brucella* (OR: 3.5; 95% CI: 1.3–9.6; P = 0.009). In addition, sweating (OR: 6; 95% CI: 1.4–26; P = 0.03) and fever (OR: 5.3, 95% CI: 2–14; P-value: 0.001) were significantly associated with *Brucella* infection (Table 4).

As illustrated in Fig. 1b, analysis of molecular findings demonstrated the presence of both single and co-infections involving *C*. *burnetii*, *Brucella* spp., *Bartonella* spp., and *T*. *whipplei*. In addition to single-pathogen detections, overlapping infections between two or more pathogens were observed, indicating polymicrobial involvement in a proportion of blood culture–negative infective endocarditis cases.

## 4 Discussion

Infective endocarditis (IE), particularly blood culture–negative infective endocarditis (BCN-IE), remains a major diagnostic and therapeutic challenge, often leading to delayed or inappropriate treatment. This difficulty is especially pronounced for slow-growing, fastidious, or intracellular pathogens that are not readily detected by conventional culture methods and may lack reliable serological markers. After prior antibiotic exposure, fastidious and unculturable bacteria represent the second most common cause of negative blood culture results [24]. Consequently, molecular diagnostic techniques have gained increasing importance in the etiological identification of BCN-IE, as emphasized by the modified Duke criteria and the European Society of Cardiology (ESC) guidelines, which highlight *C*. *burnetii*, *Bartonella* spp., *Brucella* spp., *Legionella* spp., and *T*. *whipplei* as key pathogens requiring nucleic acid–based detection [6,8].

In the present study, a substantial proportion of patients had negative blood (68.5%) and tissue cultures (53.4%), underscoring the limitations of conventional microbiological methods in this setting. The demographic and clinical characteristics of our cohort—predominance of male patients, mean age of approximately 47 years, and frequent involvement of the aortic valve—are consistent with previous studies from Iran and other regions [18]. As expected, among culture-positive cases, *Streptococcus*, *Staphylococcus*, and *Enterococcus* species were the most frequently isolated pathogens, reflecting the typical microbiological spectrum of culture-positive IE [25].

*C*. *burnetii*, the most common cause of BCN-IE worldwide, accounted for 6.9% of IE cases in this study. Diagnosis of Q fever endocarditis is often challenging because of nonspecific clinical manifestations, variable serological responses, and the frequent absence of typical valvular vegetations or inflammatory changes. These factors contribute to underdiagnosis and delayed initiation of appropriate therapy, which has been associated with increased mortality in PCR-positive patients who did not receive anti–*Coxiella* treatment [24].

An important finding of this study relates to antimicrobial treatment patterns. The most commonly prescribed antibiotics before and after surgery included vancomycin, cefepime, cefixime, gentamicin, ampicillin, and cloxacillin. According to ESC guidelines, these regimens primarily target staphylococcal, streptococcal, and enterococcal infections and are generally ineffective against fastidious intracellular pathogens. This observation suggests that empirical treatment strategies may inadequately cover atypical causes of IE, likely due to challenges in pathogen identification or underrecognition of their clinical relevance. In contrast, current ESC recommendations for BCN-IE advocate doxycycline combined with hydroxychloroquine for *C. burnetii* and *T. whipplei*, doxycycline plus gentamicin for *Bartonella* spp., and doxycycline in combination with cotrimoxazole or rifampin for *Brucella* spp. [26], highlighting the importance of accurate etiological diagnosis to guide targeted therapy.

In our cohort, *C. burnetii* infection predominantly affected relatively young patients (mean age 44 years) from urban areas, particularly Tehran and Gilan, with preferential involvement of the aortic valve. Similar patterns have been reported in Iranian studies conducted between 2016 and 2020 [18,27], although prevalence estimates varied widely (8–33%), likely reflecting differences in diagnostic strategies and study populations. The younger age distribution and urban predominance observed in Iran may represent an early warning signal for the increasing importance of Q fever in urban environments and its potential transmission through multiple routes. In contrast, studies from South Korea and other regions have reported higher prevalence rates, often affecting older patient populations [24,28].

*Brucella* spp. were detected in 8.6% of IE patients and approximately 12% of BCN-IE cases, with a mean age of 45 years. Although *Brucella* endocarditis is rare, it is clinically significant as the leading cause of mortality associated with brucellosis [29]. The prevalence observed in this study is higher than that reported in several earlier Iranian investigations, in which few or no cases were identified [30,31], suggesting possible underdiagnosis in previous studies.

Species-level analysis revealed a predominance of *B*. *melitensis*, consistent with epidemiological data indicating this species as the most prevalent and virulent in Iran and other endemic regions [32]. Given the endemicity of brucellosis in Iran and ongoing exposure risks, incorporation of molecular testing for *Brucella* spp. into routine evaluation of BCN-IE is warranted.

*Bartonella* spp. accounted for 5.6% of IE cases in this study, with *B*. *quintana* confirmed by sequencing in one case. *Bartonella* endocarditis has been reported globally and is estimated to cause 15–20% of BCN-IE cases [10,33]. Although dyspnea, fever, and sweating are non-specific clinical manifestations, their observed association with *C. burnetii* and *Brucella* spp. infections may have practical implications in patients with blood culture-negative infective endocarditis. In cases where conventional blood cultures remain negative and serological findings are unavailable or inconclusive, these clinical features may raise suspicion for zoonotic pathogens and may support earlier consideration of targeted molecular testing, including PCR-based assays, for these pathogens. However, these symptoms should not be considered diagnostic on their own and must be interpreted alongside microbiological and clinical findings.

In Iran, previous studies have documented *B. quintana* predominantly in urban provinces, particularly Tehran and Golestan [18]. The well-established association of *B. quintana* with poor socioeconomic conditions, overcrowding, and ectoparasite exposure has been described in multiple regions worldwide [10,33]. In our cohort, *Bartonella*-associated IE primarily affected patients younger than 50 years and showed a predilection for mitral valve involvement, findings consistent with reports from India and Western countries [33,34].

Phylogenetic clustering of isolate E270b within the *B*. *quintana* clade corroborates the PCR-based species identification and indicates the circulation of this clinically relevant pathogen among patients with infective endocarditis, underscoring the value of integrating molecular detection with phylogenetic analysis in culture-negative cases.

Endocarditis caused by *T*. *whipplei*, a rare cardiac manifestation of Whipple's disease, remains particularly challenging to diagnose due to its nonspecific clinical presentation and the inability of conventional microbiological methods to detect the organism [25]. As conventional microbiological methods are unable to detect this microorganism, most cases are identified incidentally through PCR analysis of excised heart valve tissue [35]. Epidemiological data suggest a higher prevalence of the disease in Europe, particularly France, Switzerland and Germany, and sporadic reports from North America. *T*. *whipplei* has been identified in 1-2.6% of cases with mean age of 55 years and symptoms of loss weight and diarrhea. Colonization usually occurs in childhood, but the development of the disease depends on genetic and immunological factors, as shown in familial cases by a decrease in Th1 and Th17 responses, as well as the human major histocompatibility complex genes DRB1∗13 and DQB1∗06 [36].

In our study, *T. whipplei* was detected in 3.0% of IE patients, with a mean age of 51 years and predominant aortic valve involvement, findings comparable to reports from Europe and North America, where prevalence rates of 1–7% have been described [8,25,35].

Although the observed prevalence in Iran appears low, this may reflect underdiagnosis, limited routine testing, or true epidemiological differences. Given the chronic course, immune evasion mechanisms, and potential for systemic involvement, increased clinical awareness and molecular testing for *T. whipplei* are essential in patients with BCN-IE [36,37].

The identification of co-infections involving multiple fastidious and intracellular pathogens further emphasizes the complexity of blood culture–negative infective endocarditis. The observed overlap among *C*. *burnetii*, *Brucella* spp., *Bartonella* spp., and *T*. *whipplei*, suggests shared or overlapping exposure routes, such as zoonotic transmission and vector-borne spread, in endemic settings. From a clinical perspective, polymicrobial involvement may contribute to diagnostic delays and suboptimal empiric antimicrobial therapy when molecular testing is not routinely implemented. These findings support current guideline recommendations advocating broad molecular investigation of excised valve tissues and reinforce the need for heightened clinical suspicion of mixed infections in BCN-IE.

The higher prevalence of these pathogens among younger individuals and urban residents may reflect changing patterns of zoonotic exposure. Possible explanations include indirect exposure through companion animals, consumption of unpasteurized dairy products, increasing mobility between rural and urban regions, and airborne dissemination of contaminated aerosols originating from livestock farms or slaughterhouses located near urban areas. Although these hypotheses cannot be directly evaluated in the present study, they warrant further investigation in future epidemiological studies.

This study has several limitations that should be acknowledged. First, its cross-sectional design precludes causal inference and limits the ability to assess temporal relationships between infection and clinical outcomes. Second, molecular analyses were performed on formalin-fixed paraffin-embedded heart valve tissues obtained after surgical intervention, which may not fully represent the entire spectrum of infective endocarditis, particularly cases managed conservatively. Third, prior antimicrobial exposure in many patients may have influenced both culture results and molecular detection rates. In addition, although species-level identification was performed for *Brucella* spp. and *Bartonella* spp., sequencing was not available for all positive samples, potentially limiting comprehensive genotypic characterization. Finally, serological data were not systematically available for all patients, precluding direct comparison between molecular and serological diagnostic approaches. Despite these limitations, the relatively large sample size and multicenter design strengthen the validity of the findings.

Furthermore, detailed epidemiological information regarding occupational exposure, animal contact, and consumption of unpasteurized dairy products was not consistently available in the medical records. Future prospective studies should systematically collect these data to enable a more comprehensive assessment of risk factors associated with zoonotic infective endocarditis.

## 5 Conclusions

This study demonstrates that major fastidious and intracellular pathogens, including *C*. *burnetii*, *Bartonella* spp., *Brucella* spp., and *T*. *whipplei*, play a clinically important role in blood culture–negative infective endocarditis in Iran. The identification of zoonotic and fastidious pathogens underscores the relevance of public health interventions, including food safety measures, livestock surveillance, and improved diagnostic awareness. The findings highlight the limitations of conventional diagnostic approaches and emphasize the critical value of molecular analysis of heart valve tissues for accurate etiological diagnosis. Integrating molecular methods into routine clinical practice can facilitate timely, pathogen-directed antimicrobial therapy, improve patient management, and reduce morbidity and mortality associated with infective endocarditis.

## CRediT authorship contribution statement

**Arezoo Dehghan:** Writing – original draft, Methodology, Investigation, Formal analysis, Data curation. **Mohammad Hossein Ahmadi:** Writing – review & editing, Supervision, Project administration, Methodology, Data curation, Conceptualization. **Saber Esmaeili:** Writing – review & editing, Supervision, Methodology, Investigation. **Mohammad Ali Boroumand:** Writing – review & editing, Methodology, Investigation. **Mahshid Hesami:** Writing – review & editing, Methodology, Investigation.

## Ethics approval and consent to participate

The study design was approved by the Ethics Committee of Shahed University (Approval ID: IR.SHAHED.REC.1403.075), and followed the statements of the Declaration of Helsinki.

## Data availability statement

The original contributions presented in the study are included in the article/supplementary material; further inquiries can be directed to the corresponding authors.

## Declaration of competing interest

The authors declare that they have no known competing financial interests or personal relationships that could have appeared to influence the work reported in this paper.

## References

[1] McHugh J., Saleh O.A. (2023). Updates in culture-negative endocarditis. Pathogens.

[2] AW Y., LC D. (2025).

[3] Tsolaki V., Zakynthinos G.E., Deskata K., Dimeas I., Parisi K., Makrygianni A., Giamouzis G., Zakynthinos E., Xanthopoulos A. (2025). Infective or non-infective endocarditis: a brief literature review based on a case report. J Clin Med.

[4] Li M., Kim J.B., Sastry B.K.S., Chen M. (2024). Infective endocarditis. Lancet.

[5] Nappi F. (2024). Native infective endocarditis: a state-of-the-art-review. Microorganisms.

[6] Delgado V., Ajmone Marsan N., de Waha S., Bonaros N., Brida M., Burri H., Caselli S., Doenst T., Ederhy S., Erba P.A. (2023). 2023 ESC guidelines for the management of endocarditis: developed by the task force on the management of endocarditis of the European society of cardiology (ESC) endorsed by the European association for cardio-thoracic surgery (EACTS) and the European association of nuclear medicine (EANM). Eur Heart J.

[7] Burban A., Słupik D., Reda A., Szczerba E., Grabowski M., Kołodzińska A. (2024). Novel diagnostic methods for infective endocarditis. Int J Mol Sci.

[8] Fowler V.G., Durack D.T., Selton-Suty C., Athan E., Bayer A.S., Chamis A.L., Dahl A., DiBernardo L., Durante-Mangoni E., Duval X. (2023). The 2023 duke-international society for cardiovascular infectious diseases criteria for infective endocarditis: updating the modified duke criteria. Clin Infect Dis.

[9] Roest H.I., Bossers A., van Zijderveld F.G., Rebel J.M. (2013). Clinical microbiology of Coxiella burnetii and relevant aspects for the diagnosis and control of the zoonotic disease Q fever. Vet Q.

[10] Wang W., Chen O., Liu W., Gan L., Li X., Ma Q., Hu X., Jian X. (2022). Coxiella burnetii and bartonella endocarditis diagnosed by metagenomic next-generation sequencing. J Clin Med.

[11] Bullard R., Olsen E., Cheslock M., Embers M. (2023). Evaluation of the available animal models for bartonella infections. One Health.

[12] Khurana S.K., Sehrawat A., Tiwari R., Prasad M., Gulati B., Shabbir M.Z., Chhabra R., Karthik K., Patel S.K., Pathak M. (2021). Bovine brucellosis–a comprehensive review. Vet Q.

[13] Dolmans R.A., Boel C.H., Lacle M.M., Kusters J.G. (2017). Clinical manifestations, treatment, and diagnosis of Tropheryma whipplei infections. Clin Microbiol Rev.

[14] AA k (2001).

[15] Howe J.R., Klimstra D.S., Cordon-Cardo C. (1997). DNA extraction from paraffin-embedded tissues using a salting-out procedure: a reliable method for PCR amplification of archival material. Histol Histopathol.

[16] Patel P.G., Selvarajah S., Boursalie S., How N.E., Ejdelman J., Guerard K.P., Bartlett J.M., Lapointe J., Park P.C., Okello J.B. (2016). Preparation of formalin-fixed paraffin-embedded tissue cores for both RNA and DNA extraction. J Vis Exp.

[17] Schneeberger P.M., Hermans M.H., van Hannen E.J., Schellekens J.J., Leenders A.C., Wever P.C. (2010). Real-time PCR with serum samples is indispensable for early diagnosis of acute Q fever. Clin Vaccine Immunol.

[18] Moradkasani S., Latifian M., Salehi-Vaziri M., Bagheri Amiri F., Mostafavi E., Ghasemi A., Esmaeili S. (2024). Molecular investigation of Coxiella burnetii, Brucella spp., Ehrlichia spp., and Borrelia spp. among patients suspected of having Crimean-Congo Hemorrhagic fever in Iran. J Infect Public Health.

[19] Hinić V., Brodard I., Thomann A., Cvetnić Z., Makaya P.V., Frey J., Abril C. (2008). Novel identification and differentiation of Brucella melitensis, B. abortus, B. suis, B. ovis, B. canis, and B. neotomae suitable for both conventional and real-time PCR systems. J Microbiol Methods.

[20] Latifian M., Mostafavi E., Broumand M.A., Amiri F.B., Mohammadi M.R., Esmaeili S. (2025). Molecular investigation of Coxiella burnetii and Bartonella in heart valve specimens of patients with endocarditis in Iran. J Infect Public Health.

[21] Kim C.M., Kim J.Y., Yi Y.H., Lee M.J., Cho M.R., Shah D.H., Klein T.A., Kim H.C., Song J.W., Chong S.T. (2005). Detection of Bartonella species from ticks, mites and small mammals in Korea. J Vet Sci.

[22] Sayyahfar S., Latifian M., Esmaeili P., Baseri N., Bagheri Amiri F., Bakhshi B., Esteghamati A., Esmaeili S. (2022). Tropheryma whipplei in the stool samples of children with acute diarrhea: a study from Tehran, Iran. BMC Infect Dis.

[23] Moravedji M., Beig M., Baseri N., Rahravani M., Latifian M., Esmaeili S. (2023). Molecular detection of Brucella abortus and Brucella melitensis in domestic ruminants and their ticks in selected areas of western Iran. Iran J Vet Res.

[24] Jang Y.R., Song J.S., Jin C.E., Ryu B.H., Park S.Y., Lee S.O., Choi S.H., Soo Kim Y., Woo J.H., Song J.K. (2018). Molecular detection of Coxiella burnetii in heart valve tissue from patients with culture-negative infective endocarditis. Medicine (Baltim).

[25] Ioannou P., Kourtidis M., Mytilinis D.O., Psyllaki A., Baliou S., Kofteridis D. (2023). Whipple's disease-associated infective endocarditis: a systematic review. Inf Disp.

[26] Fournier P.E., Thuny F., Richet H., Lepidi H., Casalta J.P., Arzouni J.P., Maurin M., Célard M., Mainardi J.L., Caus T. (2010). Comprehensive diagnostic strategy for blood culture-negative endocarditis: a prospective study of 819 new cases. Clin Infect Dis.

[27] Moradnejad P., Esmaeili S., Maleki M., Sadeghpour A., Kamali M., Rohani M., Ghasemi A., Bagheri Amiri F., Pasha H.R., Boudagh S. (2019). Q fever endocarditis in Iran. Sci Rep.

[28] Duong H.D., Do A.T.V., Bui H.T.V., Vu T.M. (2023). Molecular detection of Coxiella burnetii infection in patients with a negative infective endocarditis culture following cardiovascular surgery. World Acad Sci J.

[29] Pan S., Zhao Y., Zhou K., Chen S., Maimaitiming M., Wu J., Tuerxun M., Chong Y., Zhu J. (2024). Incidence and outcomes of brucella endocarditis in a high-prevalence area: a single-center study. J Epidemiol Glob Health.

[30] Aghamohammad S., Amirjamshidi N., Nosrati M.S.S., Mosatafavi E., Moradnejad P., Mozaffari K., Mahdieh N., Maleki M., Pasha H.R., Esmaeili S. (2023). Fastidious bacterial pathogens in replaced heart valves: the first report of Bartonella quintana and Legionella steeli in blood culture-negative endocarditis from Iran. Multidiscip Cardiovasc Ann.

[31] Mohammadi M.R., Mohabbati Mobarez A., Broumand M.A., Baseri N., Latifian M., Esmaeili S. (2025). Molecular diagnosis of infective endocarditis from culture-negative valve samples in a tertiary hospital in Iran. Microbiol Spectr.

[32] Dadar M., Alamian S., Behrozikhah A.M., Yazdani F., Kalantari A., Etemadi A., Whatmore A.M. (2019). Molecular identification of Brucella species and biovars associated with animal and human infection in Iran. Vet Res Forum.

[33] Sato S., Shapira L., Tasher D., Maruyama S., Giladi M. (2023). Molecular epidemiology of Bartonella quintana endocarditis in patients from Israel and Eastern Africa. BMC Infect Dis.

[34] Keller M., Agladze M., Kupferman T., Rich S.N., Marx G.E., Gnanaprakasam R., Kodama R., Feldmesser M., Mitchell K., Wroblewski D. (2024). Bartonella quintana endocarditis in persons experiencing homelessness, New York, New York, USA, 2020-2023. Emerg Infect Dis.

[35] Fenollar F., Célard M., Lagier J.C., Lepidi H., Fournier P.E., Raoult D. (2013). Tropheryma whipplei endocarditis. Emerg Infect Dis.

[36] Anselmo A., Rizzo F., Gervasi E., Corrent L., Ciammaruconi A., Fillo S., Fortunato A., Marella A.M., Costantini S., Baldassari L. (2025). Tropheryma whipplei and Giardia intestinalis Co-infection: metagenomic analysis during infection and the recovery Follow-Up. Infect Dis Rep.

[37] Moro L., Zavarise G., Castagna G., Pomari E., Perandin F., Piubelli C., Mazzi C., Beltrame A. (2024). Tropheryma whipplei colonization in adults and children: a prospective study. Microorganisms.

